# Cytomegalovirus establishes a latent reservoir and triggers long-lasting inflammation in the eye

**DOI:** 10.1371/journal.ppat.1007040

**Published:** 2018-05-31

**Authors:** Valentina Voigt, Christopher E. Andoniou, Iona S. Schuster, Anna Oszmiana, Monique L. Ong, Peter Fleming, John V. Forrester, Mariapia A. Degli-Esposti

**Affiliations:** 1 Immunology and Virology Program, Centre for Ophthalmology and Visual Science, The University of Western Australia, Crawley, Western Australia, Australia; 2 Centre for Experimental Immunology, Lions Eye Institute, Nedlands, Western Australia, Australia; 3 University of Aberdeen, Division of Applied Medicine, Section of Immunology and Infection, Institute of Medical Sciences, Foresterhill, Aberdeen, Scotland, United Kingdom; Cardiff University, UNITED KINGDOM

## Abstract

Recent outbreaks of Ebola and Zika have highlighted the possibility that viruses may cause enduring infections in tissues like the eye, including the neural retina, which have been considered immune privileged. Whether this is a peculiarity of exotic viruses remains unclear, since the impact of more common viral infections on neural compartments has not been examined, especially in immunocompetent hosts. Cytomegalovirus is a common, universally distributed pathogen, generally innocuous in healthy individuals. Whether in immunocompetent hosts cytomegalovirus can access the eye, and reside there indefinitely, was unknown. Using the well-established murine cytomegalovirus infection model, we show that systemic infection of immunocompetent hosts results in broad ocular infection, chronic inflammation and establishment of a latent viral pool in the eye. Infection leads to infiltration and accumulation of anti-viral CD8^+^ T cells in the eye, and to the development of tissue resident memory T cells that localize to the eye, including the retina. These findings identify the eye as an unexpected reservoir for cytomegalovirus, and suggest that common viruses may target this organ more frequently than appreciated. Notably, they also highlight that infection triggers sustained inflammatory responses in the eye, including the neural retina.

## Author summary

Cytomegalovirus (CMV) is a common viral pathogen which is highly prevalent, but does not cause clinical disease in hosts with a fully competent immune system. After infection the virus remains with the host life-long in a chronic and then latent state. Latency is thought to establish primarily in the lung and in the salivary glands, and immune privileged tissues, such as the eye and the brain are considered inaccessible to CMV unless the host is severely immunocompromised. Here Voigt et al show that following a systemic infection of immunocompetent hosts CMV accesses the eye and establishes a reservoir of latent virus in this tissue. Ongoing inflammation in the eye, including the neural retina, is then sustained long-term in the absence of viral replication. This study reveals that virally induced inflammation in immune privileged tissues may be a general phenomenon and can occur despite a fully competent immune system.

## Introduction

The concept that the eye, and other tissues in which immune privilege has been predicated, can act as a reservoir for viral infection has received increased attention recently. Ebola virus was detected in the eye of a patient who survived the initial infection and was considered fully recovered, but presented with inflammation of uveal tissues i.e. uveitis [1, 2]. Subsequently, ocular complications were noted in almost 60% of Ebola virus survivors [3]. Ocular inflammation (uveitis) has also been reported in patients with a history of dengue fever that had subsided without other complications [4]. More recently, Zika virus has been shown to affect the eye, causing severe eye disease (optic neuritis, chorioretinal atrophy) and blindness in newborns. In adults, Zika virus can induce uveitis, a finding noted recently in animal models [5].

Human CMV (HCMV) is a very common pathogen, with a worldwide sero-prevalence ranging from 40 to > 90% [6, 7]. HCMV infection is usually contracted early in life, and after an initial, generally asymptomatic infection, and partial viral control, the virus establishes a persistent and then latent infection that lasts for life [8]. Ocular inflammation has been noted in HCMV infected individuals, however, despite the presence of CMV DNA in the aqueous humor of the eye, it remains unclear whether active CMV infection, as might occur during CMV reactivation, is the primary cause of the inflammation [9]. Similarly, CMV has also been implicated in some forms of acute aseptic meningitis and encephalitis in immunocompetent patients [10], but there are no reports of live virus being cultured from cerebrospinal fluid samples.

Life-threatening complications arising from HCMV reactivation are a common occurrence in immunosuppressed individuals (reviewed in [11, 12]) and in critically ill immunocompetent patients [13] in whom the infection is associated with prolonged hospitalization and/or mortality. HCMV reactivation in immunosuppressed patients manifests as an array of clinical syndromes including pneumonitis, hepatitis and colitis [14]. CMV infection of the central nervous system (CNS) and the retina are prominent features of immune-incompetence or severe immunosuppression, as occurs in neonates [15–17], untreated AIDS patients or patients who do not respond to HAART or discontinue therapy [18], and patients with haematological malignancies and graft versus host disease [19–21].

CMVs are strictly species specific, thus, *in vivo* experimental studies using HCMV are not possible. Due to the similarities in sequence and *in vivo* pathogenesis, murine CMV (MCMV) is widely utilized as a model of HCMV infection. In this model, initial acute infection lasts less than 2 weeks. Viral titres peak at 5 days, and by 10 days the virus is cleared from the blood and most target organs. By day 14 replicating virus is only detectable in the salivary gland, where MCMV establishes a persistent infection that lasts 30–60 days depending on the mouse strain. The virus then enters a state of latency, where replicating virus is not detectable [22]. Importantly, the mouse model of CMV infection does not require manipulation of the host. Unlike many other viruses, MCMV, a natural mouse pathogen, provides a unique model to study a medically important virus *in vivo* after infection of its biological host. Indeed, preclinical mouse models of CMV infection have provided valuable insights into the immunobiology of allogeneic haematopoietic stem cell or bone marrow transplantation, with much of the information obtained from these models already translated into clinical practice [23].

The relevance of inflammation at immune privileged sites, including the CNS, is increasingly appreciated. However, it remains unclear what the triggers of such inflammation are, and how sustained these responses may be. Several models have been used to study the etiology and pathogenesis of CMV infection in the eye, including the neural retina, *in vivo*. These have relied primarily on intraocular injection of virus [24], infection of mice in which the blood-retinal barrier has been disrupted [25], or infection of severely immunosuppressed mice [26]. By contrast, the impact of CMV on ocular compartments, including the neural retina, in immunocompetent hosts following systemic infection has been poorly characterised.

Here, we demonstrate that following systemic infection of immunocompetent mice, (a) MCMV causes a chronic uveitis beginning within 48 hours and lasting indefinitely, even after viral replication is no longer detectable, (b) MCMV infects the anterior segment of the eye, with replicating virus detected throughout the uveal tract (iris, ciliary body and choroid), but not the retina, for up to 10 days post infection and (c) MCMV can be cultured from explants of uveal tissues months after the initial infection has been controlled. Notably, persistent inflammatory changes, including infiltration of virus-specific CD8^+^ T cells and the development of tissue-resident memory cells (T_RM_) were noted in the retina despite the absence of detectable viral replication in this compartment. Thus, the eye is both a site of viral replication and viral latency for a common viral pathogen. These findings highlight the need to consider the ocular microbiome when investigating not only pathogenic processes, but also ‘normal’ physiological situations, as these viruses are common ‘residents’ in the eye. The data also demonstrate that immunosuppression is not a prerequisite for CMV-mediated eye disease and that severe and sustained inflammation is triggered by this infection.

## Results

### Systemic MCMV infection of immunocompetent hosts results in ocular inflammation

**Fig 1:**
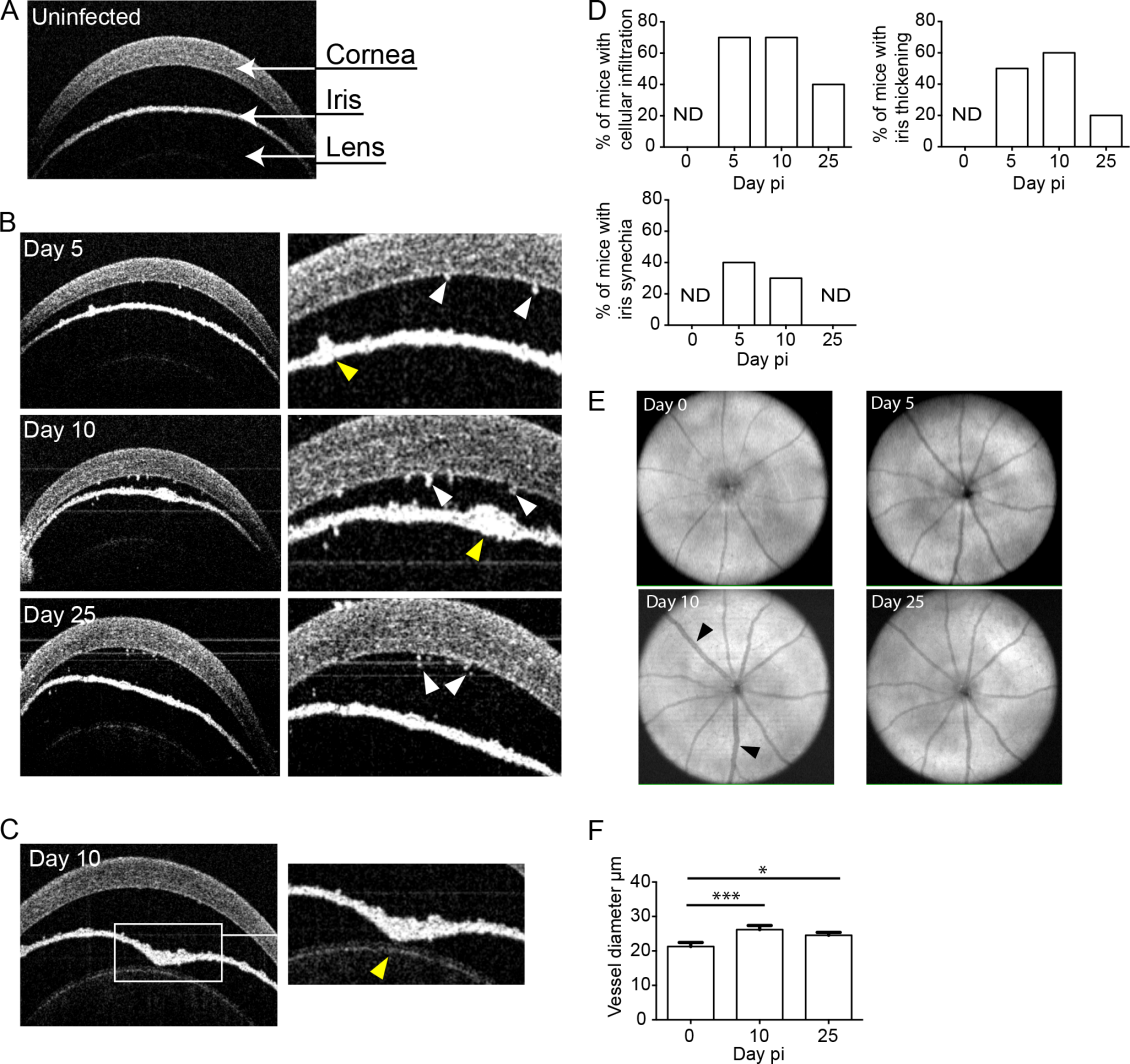
Pathological changes in the eye after systemic MCMV infection. **(A)** SD-OCT image of the anterior segment of the eye from an uninfected mouse. **(B)** Cross-sections of the anterior chamber from mice infected with MCMV at the indicated times pi. Cellular deposits (white arrows), vessel dilation and iris thickening (yellow arrows). **(C)** A representative image demonstrating iris bombé, and the formation of synechia (yellow arrow). **(D)** The frequency of mice displaying the indicated pathological features following MCMV infection was quantified where n = 10 mice for each time point from at least 3 independent experiments. ND = not detected. **(E)** SD-OCT fundus images of the retina at the indicated times pi. Black arrows indicate retinal vessel enlargement (dilation and calibre variation). **(F)** Vessel diameter was measured at the indicated times pi with mean ± standard error of the mean (SEM) plotted, where n≥8 eyes (* P = 0.035; *** P = 0.0006).

To evaluate the impact of systemic MCMV infection on the eye we used adult BALB/c mice that are genetically susceptible to MCMV infection. Mice were systemically infected with MCMV by intraperitoneal injection (IP) and spectral domain optical coherence tomography (SDOCT) was used to monitor the impact of infection on the eye. SDOCT is a non-invasive technique that uses near-infrared light to generate high-resolution cross-sectional images of the eye. The eye can be grossly divided into two major anatomical components: the anterior and posterior segments. The anterior segment comprises the cornea, iris, ciliary body and lens; the posterior segment includes the vitreous, retina, choroid and optic nerve. A fluid (aqueous humor) filled chamber (anterior chamber) separates the cornea from the iris. A representative image of the anterior segment from an uninfected mouse is shown, with the relevant components identified (**Fig 1A)**. As early as day 5 post-infection (pi), cells were visible in the anterior chamber, and cellular deposits were observed on the corneal endothelium (**Fig 1B, white arrows**); these became markedly obvious by day 10 pi, and were still apparent at day 25 pi (**Fig 1B, white arrows)**. Patchy iris thickening and vessel dilation were also evident (**Fig 1B, yellow arrows**). Synechiae, a condition where the iris adheres to the lens, were frequently observed after infection (**Fig 1C, yellow arrow**). Formation of synechiae blocks the flow of aqueous humor resulting in an increase in intraocular pressure that manifests as forward bowing of the iris (iris bombé) (**Fig 1C**). The frequency of the various pathological features observed after MCMV infection was quantified at several times pi (**Fig 1D**). Pathological changes associated with MCMV infection were highest at day 5–10 pi and decreased by day 25 pi. Retinal imaging microscopy identified vascular changes from day 10 pi (**Fig 1E, black arrows**) with significant increases in the diameter of the retinal vessels (vessel dilatation and calibre variation) observed after infection (**Fig 1F**).

**Fig 2:**
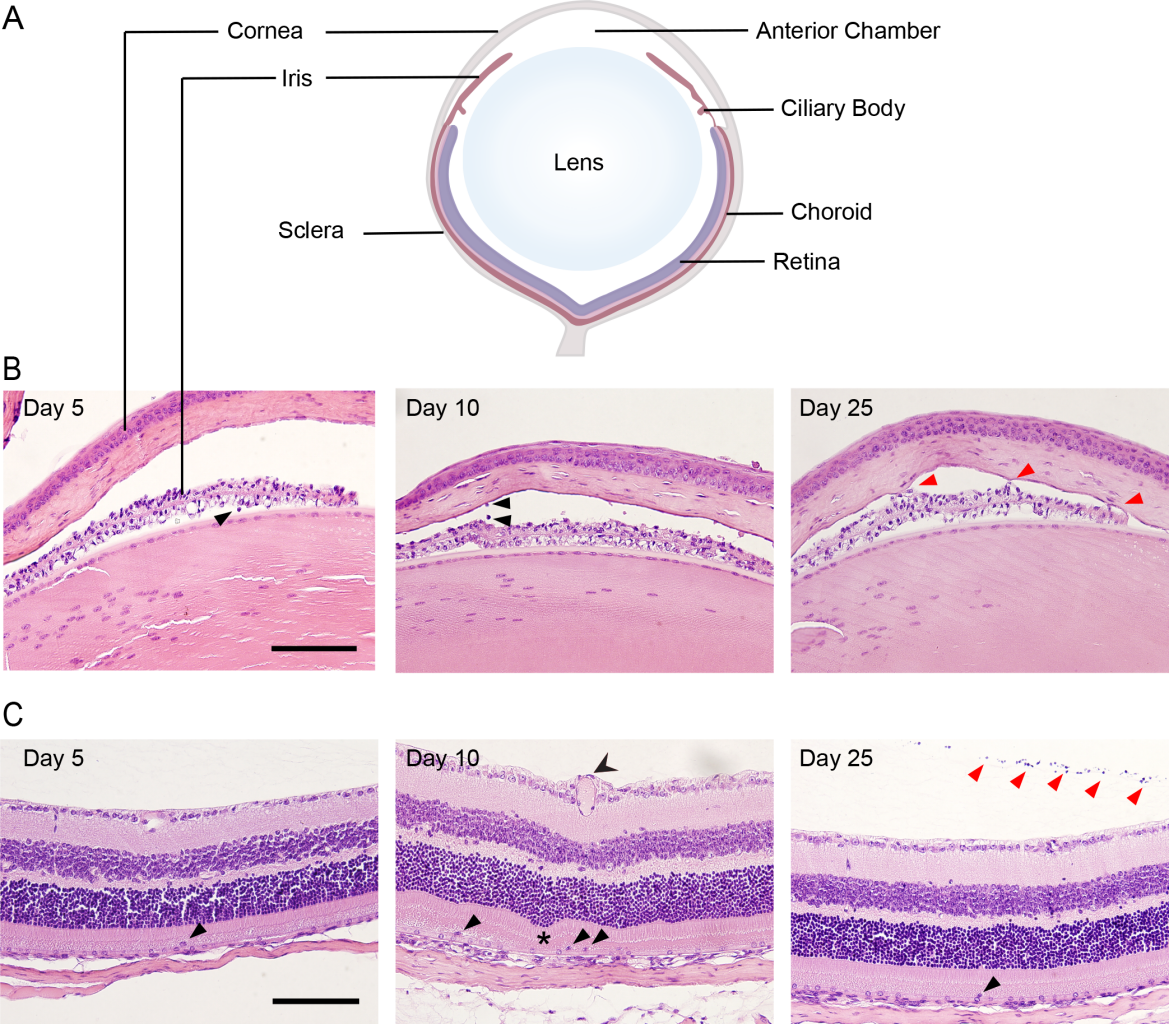
MCMV infection causes inflammation in the iris and retina. **(A)** Schematic diagram with the major compartments of the eye labelled. **(B)** Sections of the anterior chamber at the indicated times pi were stained with haematoxylin and eosin. Thickening of the iris was evident at day 5. Cellular infiltrates and keratic precipitates were present after infection (black arrows). Synechia (adherence of the iris to the cornea) was frequently observed (red arrows). **(C)** Haematoxylin and eosin stained sections of the retina show normal structure at day 5 pi. Enlarged vessels (two tailed arrow head), folds in the retina (asterisk) and infiltrating cells were present (black arrows) at day 10pi. At day 25 pi infiltrating cells within the retina (black arrow heads) and the vitreous (red arrow heads) were evident. Scale bar: 100 μm. Results are representative of those from 5 mice per time-point.

Histological analysis was undertaken to evaluate the extent of inflammation and potential pathology in various eye compartments. The major eye compartments are schematically depicted (**Fig 2A**). Inflammatory changes were noted in the anterior chamber from day 5 to day 25 pi, including cell infiltration (**Fig 2B, black arrows**) and synechiae **(Fig 2B, red arrows**). The retinal structure was also affected following infection; at day 10 pi retinal vessel enlargement (**Fig 2C, double tailed arrow**) and occasional retinal folding (**Fig 2C, asterisk**) were observed, together with inflammatory cells in the subretinal space (**Fig 2C, black arrows**). Mild vitritis (**Fig 2C, red arrows**) and rare inflammatory cells in the subretinal space were present at day 25 pi (**Fig 2, black arrow**).

### Systemic MCMV infection of immunocompetent hosts results in ocular infection

**Fig 3:**
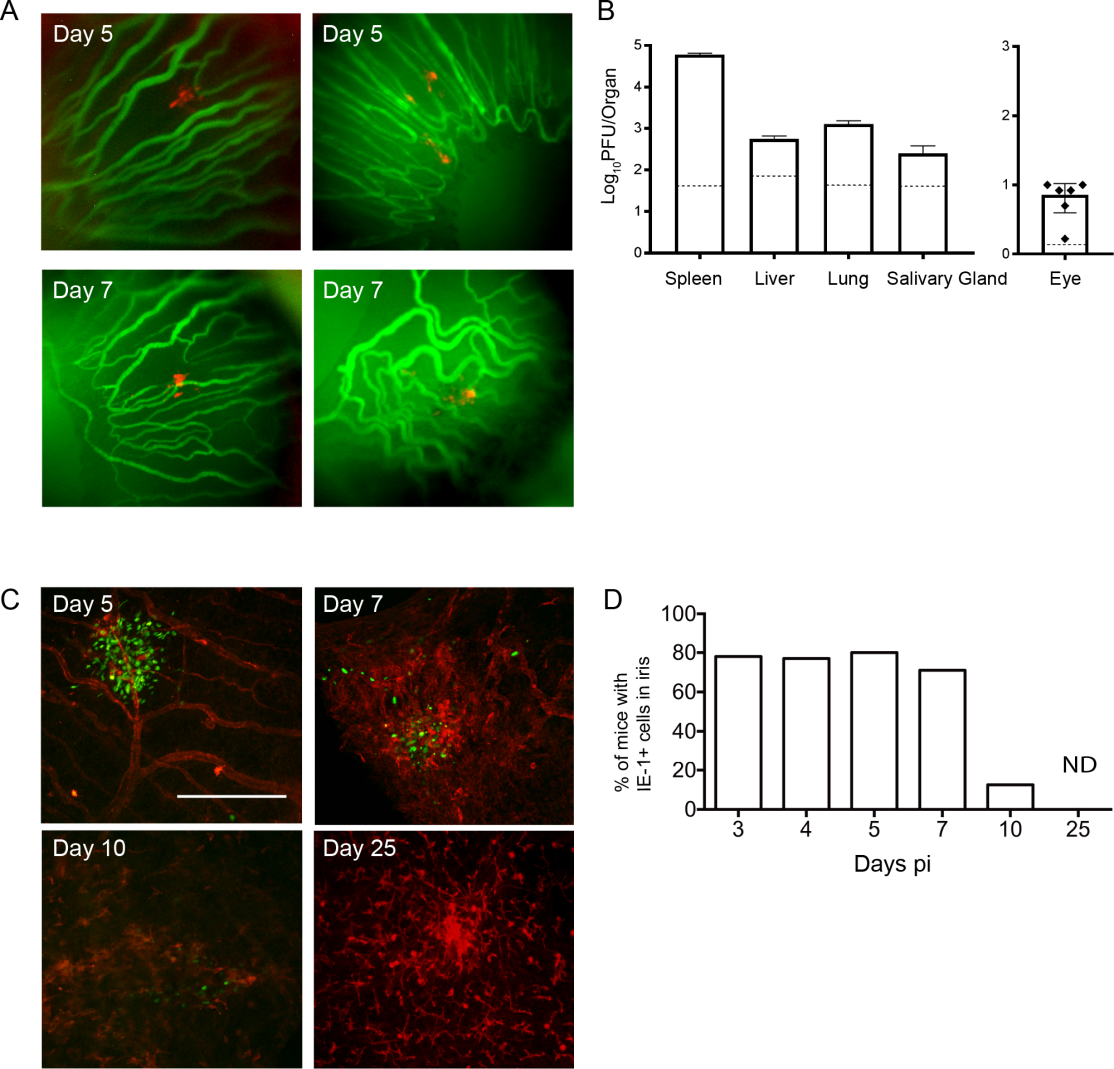
MCMV infects and replicates within the eye after systemic infection. **(A)** Mice were infected with MCMV-mCherry, and injected with fluorescein prior to *in vivo* imaging. The iris was assessed using a Micron IV imaging microscope with the vessels staining green and red fluorescence indicating MCMV-infected cells. **(B)** The indicated organs were removed from MCMV infected mice at day 5 pi, homogenized, and MCMV titres determined by plaque assay. The dashed line represents the limit of detection for individual organs. Mean ± SEM are plotted, where n≥5. **(C)** Whole-mounts of the iris were prepared at various times pi and stained with antibodies directed against MHC-II (red) and the viral antigen IE1 (green). Scale bar: 200 μm **(D)** The percentage of mice with detectable MCMV in the iris at various times pi is shown where n≥ 9 for each time point. ND = not detected.

Whether MCMV can infect and be detected in the eye after systemic infection of immunocompetent hosts is unclear. To address this question and determine whether the virus is present in the eye, we infected mice with a recombinant MCMV engineered to express the fluorescent protein mCherry, that aids the identification of infected cells. At various times pi, mice were injected with fluorescein prior to analysis to identify blood vessels. mCherry-positive cells were detected *in vivo* in the iris at day 5 and 7 pi (**Fig 3A**). The majority of mCherry positive cells were detected outside the vasculature, indicating that MCMV had infected the iris tissue. To extend this finding, eyes, along with other target organs, were harvested at day 5 pi and homogenates prepared. Infectious viral loads within organs were measured by plaque assay, and significant levels of virus were detected in organs such as spleen, liver, lungs and salivary glands (**Fig 3B**). Importantly, infectious MCMV was also reproducibly detected in eye homogenates (**Fig 3B**) indicating that the virus can effectively replicate in some compartments of the eye.

Eye whole-mounts provide important topographic information, including the distribution and morphology of infected cells in specific compartments of the eye. Whole-mounts of the eye were therefore prepared and stained with antibodies to the viral antigen IE1, to identify virally infected (IE1^+^) cells *in situ* and to distinguish between virus directly infecting ocular tissue and virus trafficking within the eye vasculature. At day 5 pi, foci of infected cells were readily detected in the iris with focal distribution around vessels (**Fig 3C**). The number of infected cells detected within the iris decreased thereafter, with no infected cells detectable by day 25 pi (**Fig 3C**). In order to determine if the anterior segment is a prominent target for MCMV infection, whole-mounts were prepared from a large cohort of mice infected in multiple independent experiments. This analysis indicates that MCMV infection within the iris occurred in approximately 80% of immunocompetent mice systemically infected with MCMV (**Fig 3D**). The percentage of mice with an active MCMV infection in the iris began to decrease by day 7 pi, with the infection no longer detectable in this compartment by day 25pi (**Fig 3D**).

**Fig 4:**
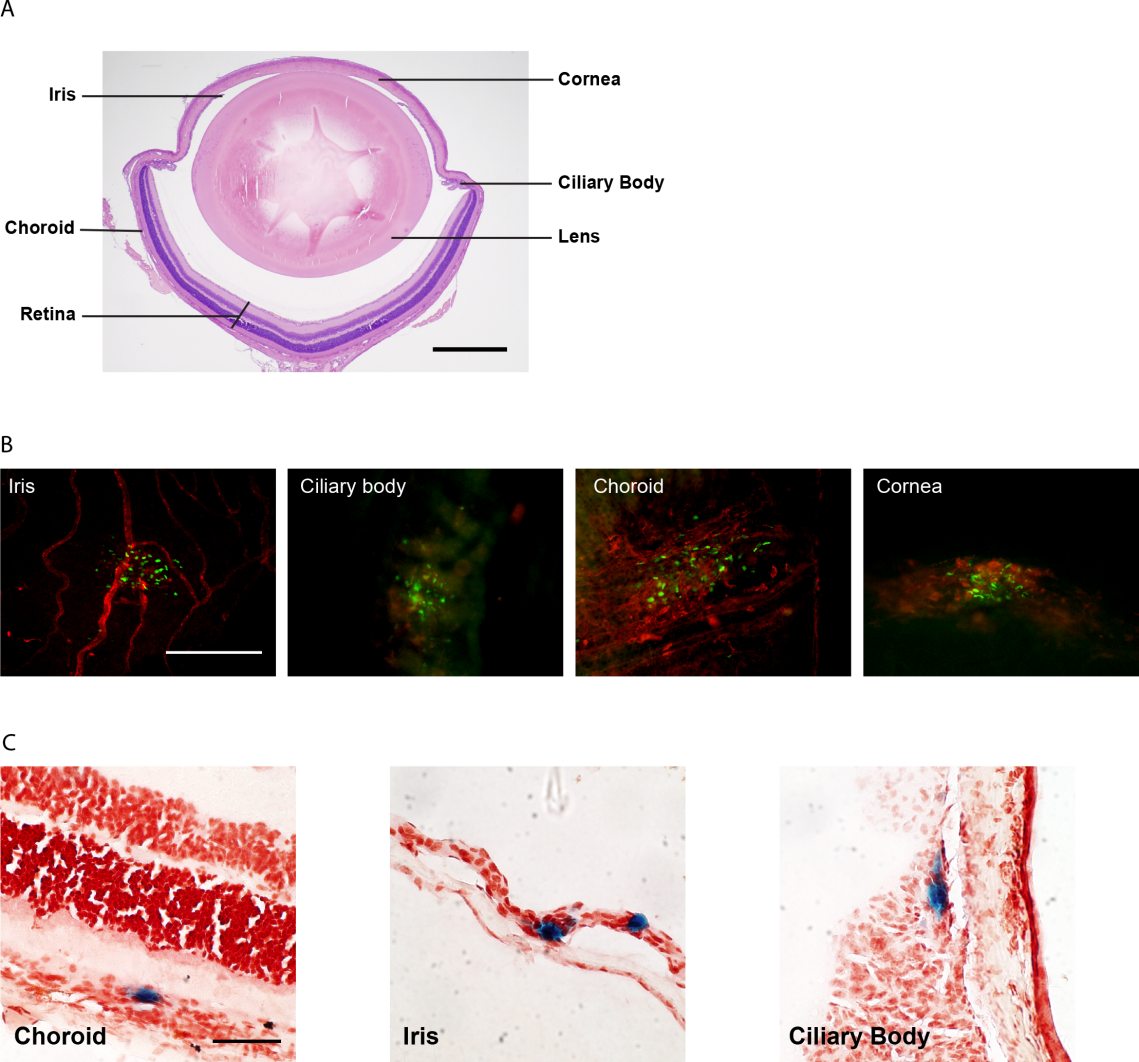
Defining the compartments of the eye infected by MCMV. **(A)** Haematoxylin and eosin stained section of an uninfected mouse eye with the major compartments labelled. Scale bar: 500 μm. **(B)** Eyes were removed from MCMV-infected mice at day 5 pi, components of the eye dissected and whole-mounts prepared. The samples were stained with antibodies against MHC-II (red) and IE1 (green—for viral antigen) and microscopy performed. Scale bar: 200 μm. Results are representative of at least 5 independent experiments with n≥20. **(C)** Mice were infected with MCMV-LacZ and eyes removed at day 5 pi. Frozen tissue sections were prepared and stained with x-gal to identify virally infected cells (blue) and counterstained with neutral red to identify cell nuclei. Scale bar: 50 μm.

The capacity for MCMV to infect other anatomical compartments within the eye (**Fig 4A**) was then assessed. Whole-mounts were prepared at day 5 pi and stained with antibodies specific for the viral IE1 protein. In addition to the iris, foci of infected cells were detected in the ciliary body, choroid and cornea (**Fig 4B**), while infection of the retina could not be detected by this method. To consolidate these findings and localise virus within the distinct compartments of the eye, mice were infected with MCMV-LacZ, a recombinant virus engineered to express β-galactosidase. Tissue sections of eyes from mice infected with MCMV-LacZ confirmed that multiple compartments of the eye harbour MCMV. Specifically, virally infected cells were detected in the choroid, iris and ciliary body (**Fig 4C**). Together these data clearly demonstrate that, following systemic infection, MCMV gains access to select compartments of the eye and is capable of effectively replicating within these sites.

### MCMV infects multiple cell types in the iris

**Fig 5:**
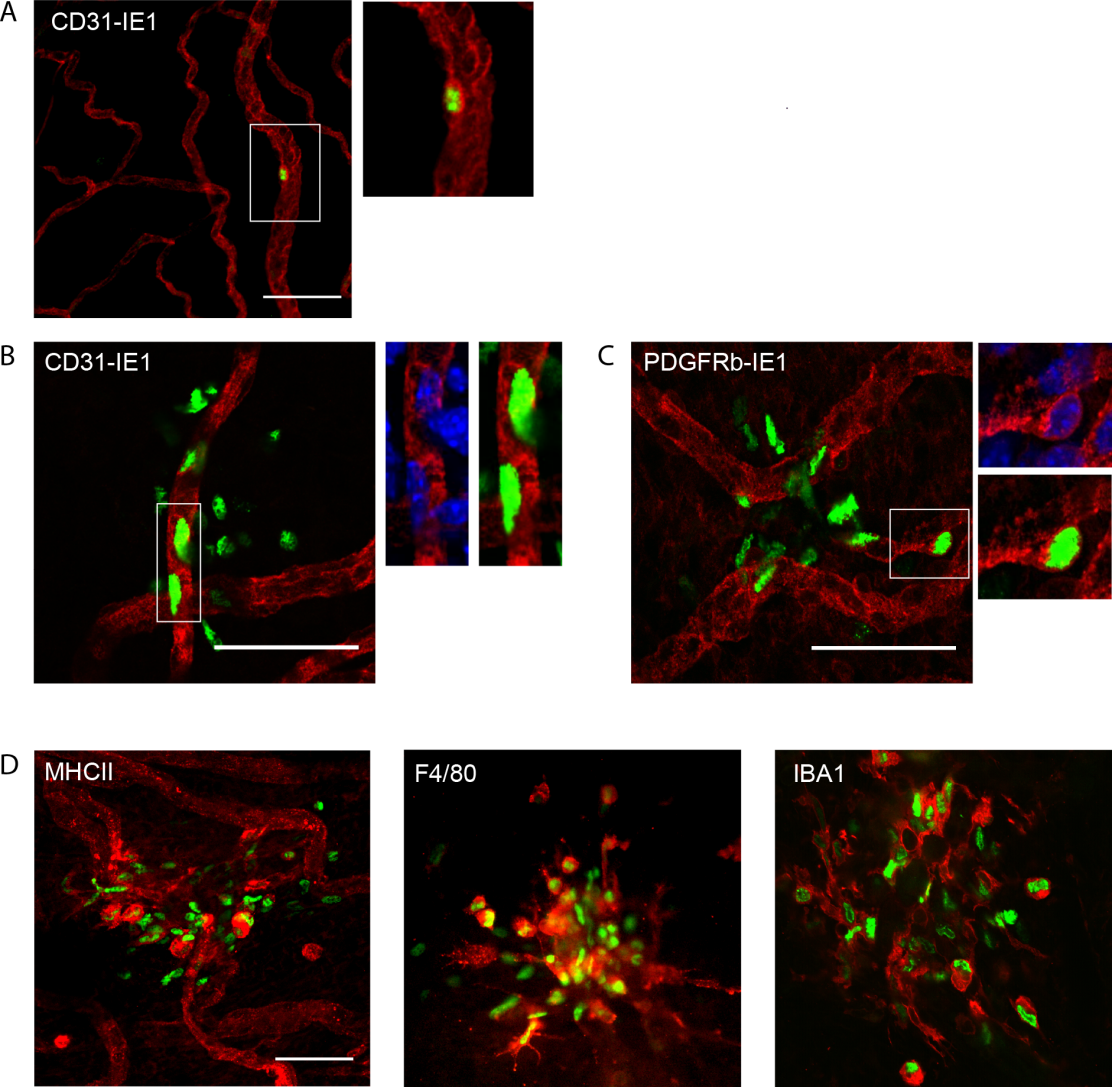
Characterisation of cells infected by MCMV within the iris. **(A)** Wholemounts of the iris were prepared at 24 hr pi and stained with antibodies to CD31 (red) and the IE1 viral antigen (green). Iris whole-mounts at day 2 pi were stained with anti-IE1 (green) and **(B)** anti-CD31 or **(C)** anti-PDGFRβ antibodies. Blue = DAPI nuclear stain. **(D)** Wholemounts of the iris at day 5 pi were stained with anti-IE1 (green) along with antibodies directed against the indicated cell surface markers (red). Anti-IE1 detects a nuclear antigen. Scale bar: 50 μm. Results are representative of at least 3 independent experiments with n≥9.

MCMV is known to be capable of infecting a wide array of cell types, including endothelial cells, macrophages and fibroblasts. To define the nature of the ocular cells targeted by MCMV, phenotyping of infected cells in the eye was performed. Given that MCMV infection was most prominent in the iris, this tissue was selected for analysis. Iris whole-mounts were stained with anti-IE1 antibodies (to detect viral antigen), in combination with cell lineage specific antibodies and analysed by microscopy. Infection, denoted by nuclear IE1 staining, was detected in individual CD31^+^ vascular endothelial cells as early as 24h pi (**Fig 5A**) with clusters of infected endothelial cells detected by day 2 pi (**Fig 5B**). In addition to endothelial cells, MCMV-infected PDGFRβ^+^ pericytes were detected from day 2 pi (**Fig 5C**), indicating that the virus can exit the vasculature and infect surrounding tissue. As the infection proceeded, in addition to endothelial cells and pericytes, virally infected, IE1^+^ cells were found to express MHC-II and macrophage markers (F4/80 and IBA1) (**Fig 5D**). Thus, vascular endothelial cells within the iris are initially infected by MCMV, with the virus then spreading to pericytes and infiltrating monocytes within the iris as the infection proceeds.

### Characterisation of inflammatory cells infiltrating ocular structures following systemic MCMV infection

**Fig 6:**
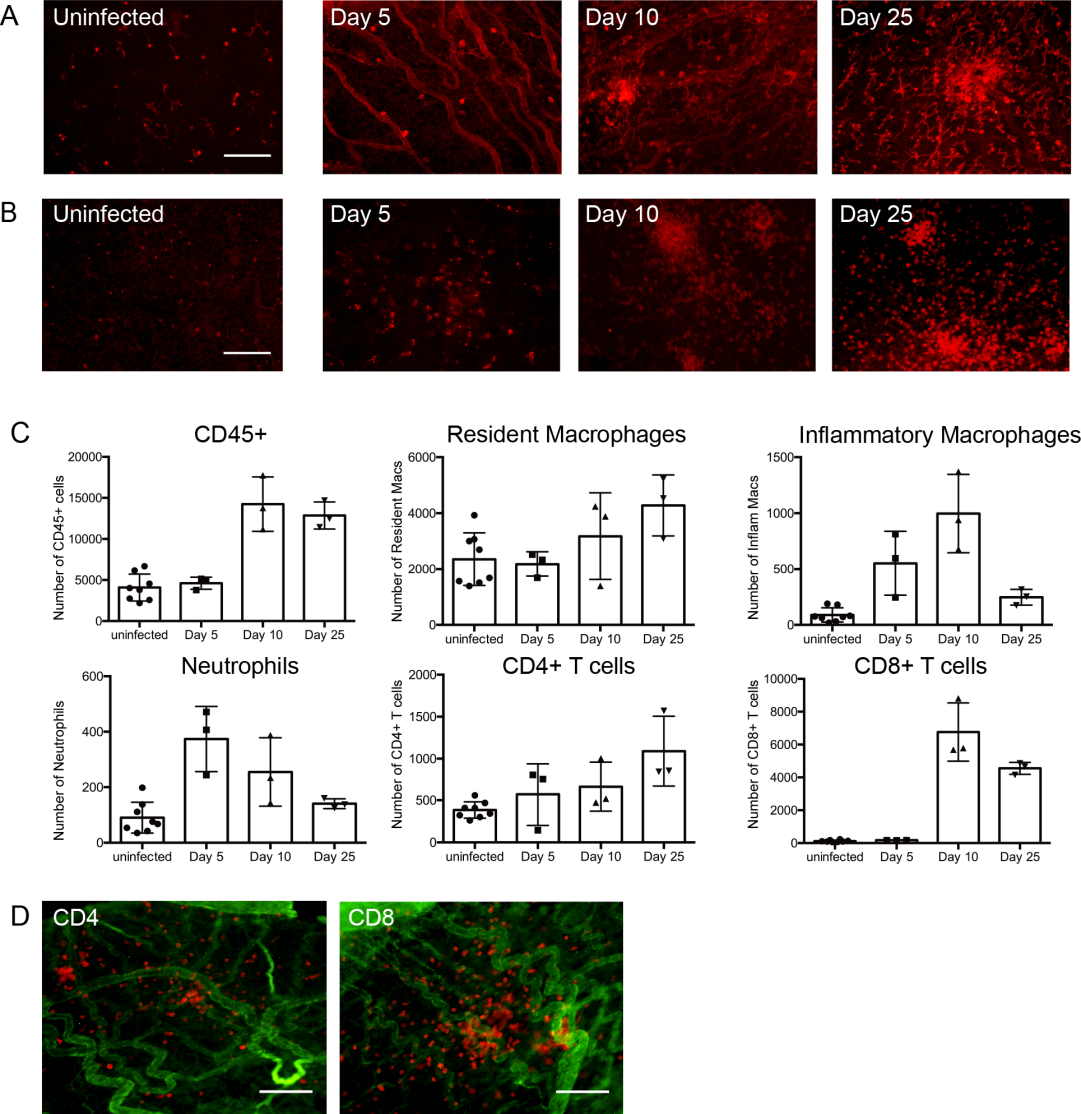
Leukocytes and lymphocytes, including T cells, infiltrate the iris after systemic MCMV infection. Whole- mounts of the iris at the indicated time points pi were prepared and stained with **(A)** anti-MHC-II or **(B)** anti-CD45 antibodies. **(C)** Mice were infected with MCMV, and at the indicated times pi single cell preparations of the iris prepared. Iris samples were pooled from 2–5 mice. The resulting pooled samples were stained with lineage specific antibodies and analysed by flow cytometry. The percentage of cells in specific populations are graphed as mean ± SEM where samples represent n≥10 mice for each time point from 3 independent experiments. **(D)** Iris whole-mounts from mice at day 25 pi were stained with collagen IV (green) and CD4 (left panel) or CD8 (right panel) antibodies (red). Scale bar: 100 μm.

The results of the OCT analysis indicated systemic MCMV infection causes inflammation in some compartments of the eye. Eye whole-mounts were used to characterize changes in the distribution of cellular components, including cells that may be infiltrating specific compartments of the eye after infection. Normal uveal tissue contains a rich population of MHC-II^+^ dendritic cells (DC) and dendritiform macrophages, while the vasculature is MHC-II^lo^ or negative ([27] and **Fig 6A, uninfected**). At day 5 pi, expression of MHC-II on the vessels within the iris was significantly increased, with expression returning to baseline by day 25 pi (**Fig 6A**). In addition, by day 10 pi an increase in the number of MHC-II^+^ cells in the iris stroma was apparent (**Fig 6A**), a finding consistent with our previous data showing that IBA1^+^ macrophages infiltrate the iris after MCMV infection [28]. These cells persisted to day 25 pi, by which time they were found to form large clusters (**Fig 6A**). To confirm these findings and detect leukocytes infiltrating the iris, whole-mounts were also stained with anti-CD45 antibodies. An increase in the number of CD45^+^ cells was apparent at day 5 pi with large clusters of CD45^+^ cells still evident in approximately 40% of the infected mice at day 25 pi (**Fig 6B)**, a time when virus is no longer detectable at this site. Quantitative analysis of the cellular infiltrate in the iris was performed by flow cytometry to better define the cells infiltrating this site in response to systemic MCMV infection. The leukocytes in an uninfected iris consisted predominantly of resident macrophages, with a small number of CD4^+^ T cells present, a finding consistent with previous work ([27] and **Fig 6C**). At day 5 pi an influx of inflammatory monocytes and neutrophils was evident, with no significant change in the proportion of other cell lineages observed (**Fig 6C**). At day 10 and 25pi, a substantial increase in the number of CD45^+^ cells localised to the iris was evident, with CD8^+^ and CD4^+^ T cells being the major infiltrating cell types (**Fig 6C**). MCMV infection did not have any significant impact on the number of B cells or NK cells localised to the iris. In order to confirm that the observed changes in T cell numbers reflected infiltration of the iris rather than an increase in T cells within the vasculature, whole-mounts from mice at day 25 pi were prepared. Staining of iris whole-mounts with anti-CD4 or anti-CD8 antibodies clearly demonstrated that these lymphocytes have exited the vasculature and localise to iris tissue (**Fig 6D**). Together, these data establish that systemic infection with MCMV induces a strong inflammatory response in the anterior segment of the eye.

### Sustained inflammatory responses in the retina following systemic MCMV infection

In contrast to the iris, and in agreement with previous findings [28], we did not detect discernible replicating MCMV in the retina. Retinal tissue was assayed for the presence of viral genome and viral transcripts at the peak of infection (day 5 and day 9), when virus was clearly identified in other eye compartments both by this method (**S1 Fig**), as well as by plaque assay (**Fig 3B**) and immunofluorescence analyses for viral antigen (**Figs 3, 4 and 5**). We were unable to detect viral genome, replicating virus or viral antigens in retinal tissue.

**Fig 7:**
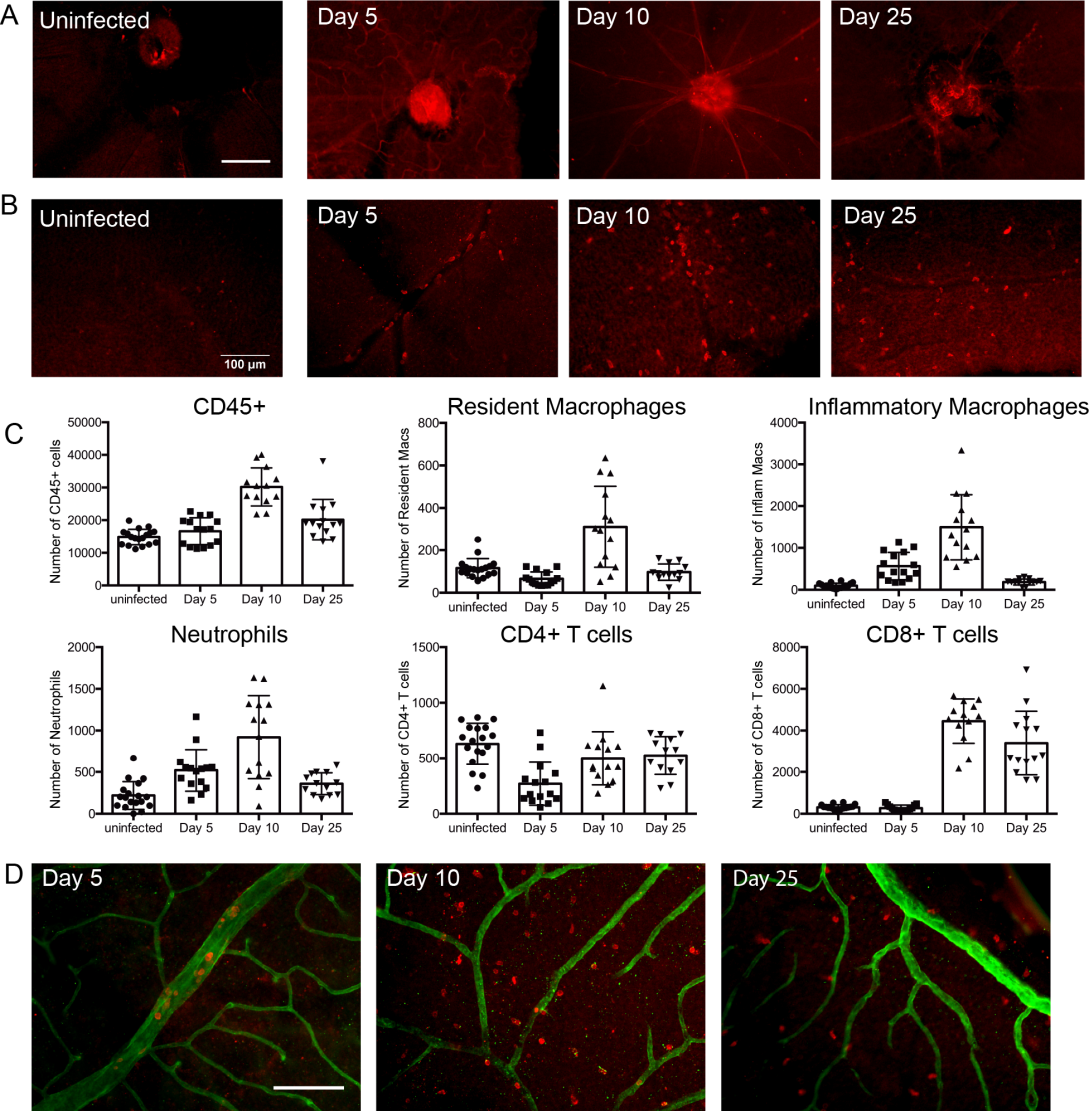
Leukocytes and lymphocytes, including T cells, infiltrate and accumulate in the retina after systemic MCMV infection. Retinal sections were stained with **(A)** anti-MHC-II or **(B)** anti-CD45 antibodies. **(C)** Mice were infected with MCMV and at the indicated times pi the retina isolated. Single cell preparations from individual retina samples were stained with lineage specific antibodies and analysed by flow cytometry. The percentages of cells in specific populations are graphed as mean ± SEM where n≥10 mice for each time point from 3 independent experiments. **(D)** Retinal whole-mounts at the indicated times pi were stained with collagen IV (green) anti-CD45 (red) antibodies. Scale bars: 100 μm.

Despite the absence of actively replicating virus at this site, the inflammatory response to systemic MCMV infection was exuberant, and similar to that observed in the iris, where replicating virus and viral antigen were detected. In uninfected mice, the retina is devoid of leukocytes and no significant MHC-II staining is evident (**Fig 7A and 7B uninfected**). Following MCMV infection, increased expression of MHC-II was observed on vessels within the retina (**Fig 7A**) and CD45^+^ leukocytes were also detected in the retina (**Fig 7B**). Flow cytometric analysis revealed that neutrophils and monocytes represent the bulk of the infiltrating cells early after infection, with CD8^+^ T cells becoming the predominant population by 25 pi (**Fig 7C**). Staining of retina whole-mounts confirmed that CD45^+^ leukocytes had exited the vasculature and entered the retina (**Fig 7D**). Thus, despite there being no detectable MCMV within the retina, leukocytes and lymphocytes infiltrate this compartment after systemic infection and, remarkably ongoing inflammation is noted, even though the host is immuno-competent.

### MCMV infection leads to the formation of tissue resident memory T cell subsets in ocular tissues, including the retina

**Fig 8:**
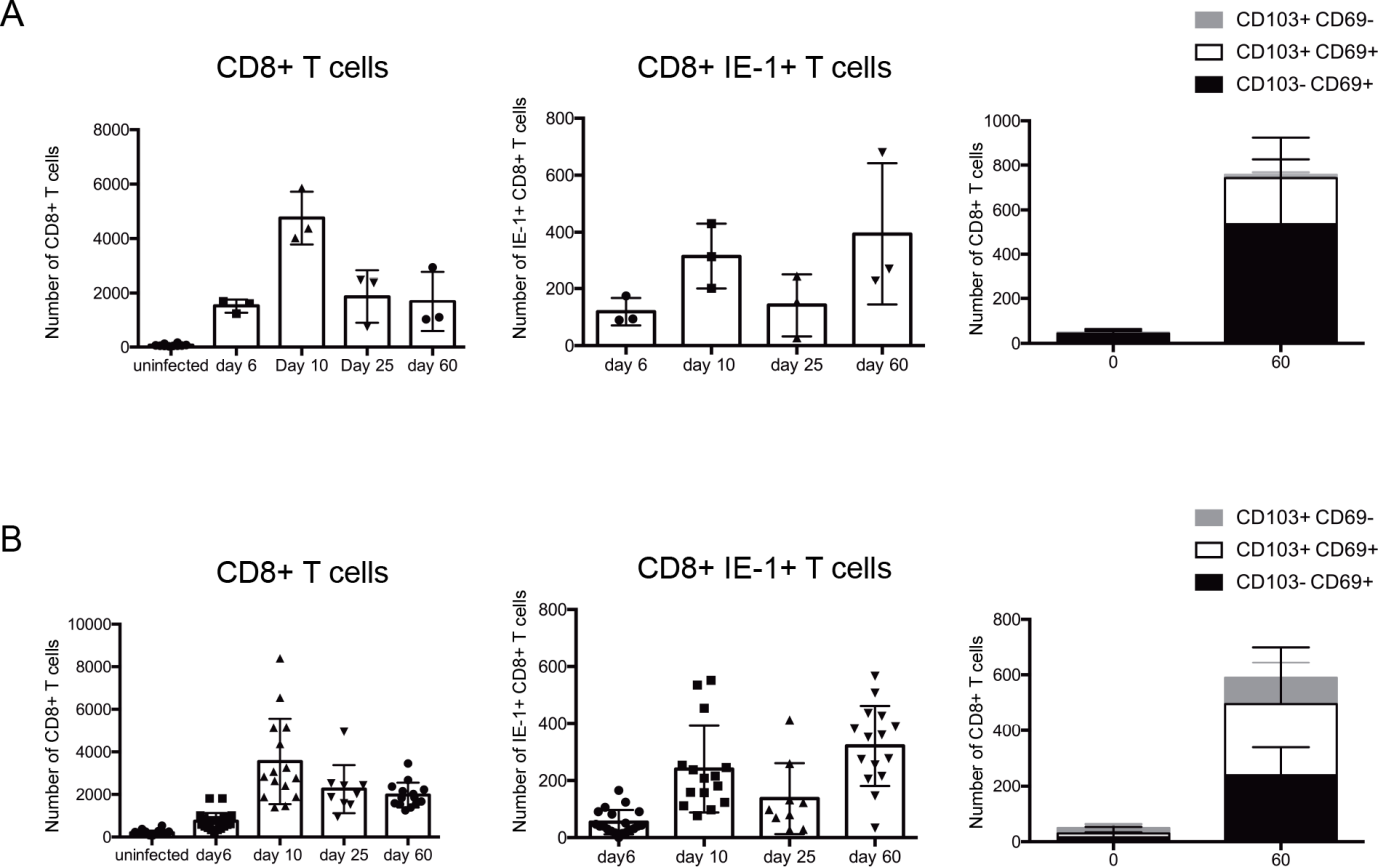
Virus-specific CD8^+^ T cells infiltrate and accumulate in the iris and retina after systemic MCMV infection and form tissue resident memory (T_RM_) populations. Mice were infected with MCMV, and at the indicated times pi single cell preparations were prepared and stained for T cell markers. (A) The number of CD8^+^ T cells, CD8^+^ IE1^+^ antiviral T cells and CD8^+^ IE1^+^ CD103^+/-^ CD69^+/-^ T cells are graphed as mean ± SEM for (**A**) the iris and (**B**) the retina. Samples represent n = 6 for uninfected mice, and n = 15 for MCMV infected mice for each time point from 3 independent experiments. Iris samples were pooled from 5 mice.

Having demonstrated that systemic infection leads to ongoing inflammatory responses both in the iris and in the retina, we examined whether these responses included virus-specific CD8^+^ T cells, a population essential for the control of acute infection and postulated as critical to prevent viral reactivation. The number of CD8^+^ T cells increased over the course of MCMV infection in both the iris and the retina (**Fig 8A and 8B**), as did the number of virus-specific CD8^+^ T cells which were detected using MHC-I tetramers for MCMV IE1 (**Fig 8A and 8B**). In BALB/c mice, which is the model used in our studies, viral latency is established by day 40 pi, when viral replication is not detected in any of the target organs, including the salivary gland [29]. We therefore extended our analyses to day 60 pi to examine inflammatory responses during viral latency. Non-recirculating T_RM_ represent long-lived memory T cells that reside in tissues well after initial antigen encounter [30]. T_RM_ typically express CD69 and/or CD103; following MCMV infection CD8^+^ T cells with a T_RM_ phenotype were detected in both the iris (**Fig 8A**) and retina at day 60 pi (**Fig 8B**). Thus, a pool of tissue resident memory T cells is established in the eye after systemic MCMV infection.

### MCMV establishes a latent infection in the eye

**Fig 9:**
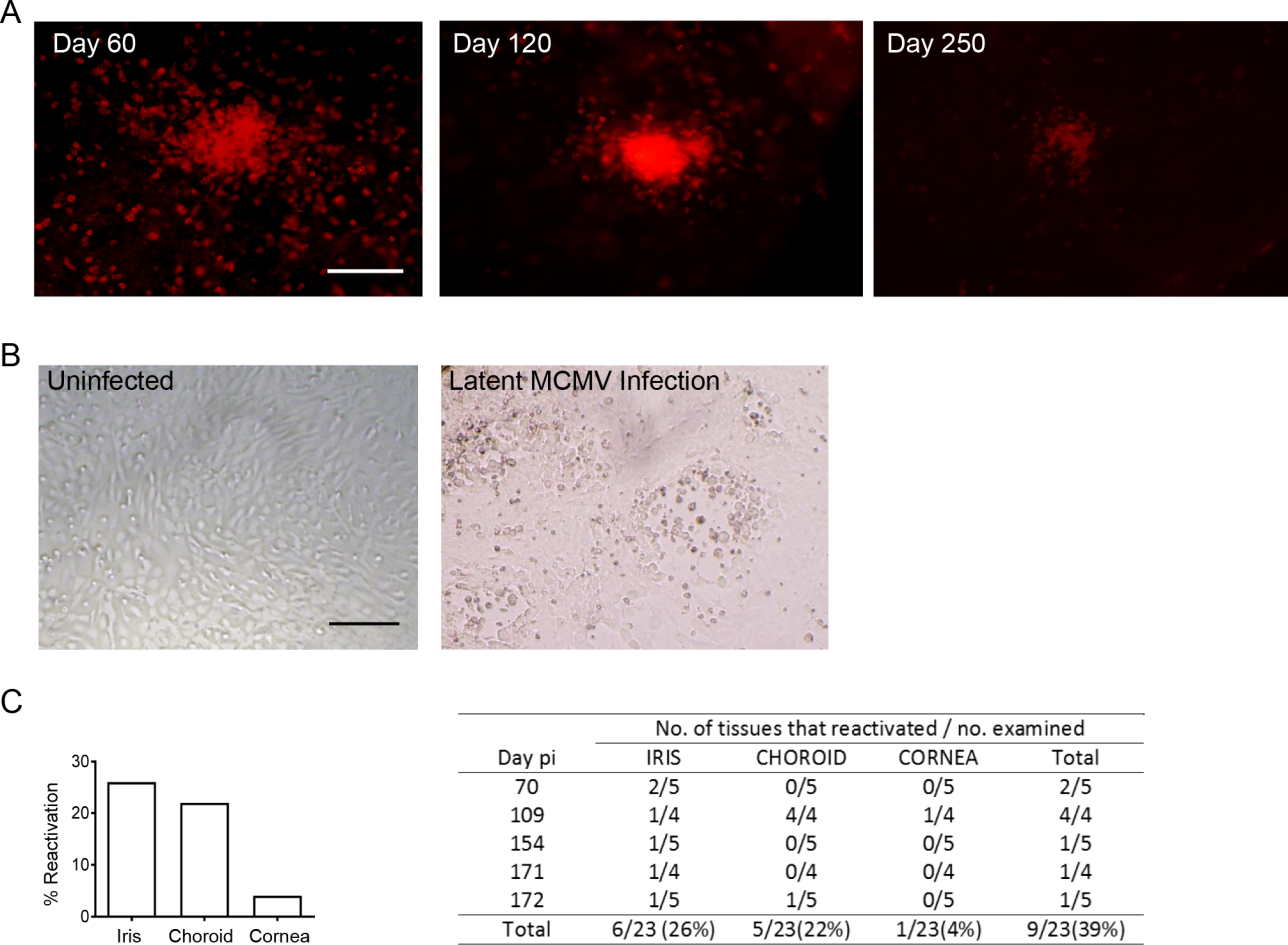
MCMV establishes a latent infection in ocular tissue. **(A)** At the indicated times pi iris whole-mounts were stained with anti-CD45 antibodies. **(B)** A representative image of the cellular monolayer of iris explant cultures established from an uninfected mouse (left panel) or a latently infected mouse (right panel). The culture established from a latently infected mouse shows foci of viral replication and cytomegalic cells characteristic of MCMV infection. Scale bar: 200 μm. **(C)** Percentage of cultures where MCMV reactivation was detected. (n = 23 mice). The table on the right shows the frequency of viral reactivation in specific compartments of the eye from individual mice.

Clusters of CD45^+^ leukocytes were found in the iris at day 25 pi (**Fig 6B)**. Since MCMV infection is rapidly controlled at this site, we assumed that the aggregates of leukocytes would disperse after resolution of the infection. Surprisingly, large clusters of CD45^+^ cells resembling granulomas were still detectable at later times after infection, and as late as day 250 pi (**Fig 9A**). No IE1^+^ (virally-infected) cells could be detected in these sections, indicating that this inflammation was occurring in the absence of active viral replication. The persistence of leukocytes at this site could signify that a latent infection has been established. To test this possibility, mice were infected with MCMV for at least 70 days, a time point where no active viral replication is detectable. Eyes were then isolated from latently infected mice, the cornea, iris and choroid dissected, and explant cultures established. The cultures were assayed for the presence of MCMV by plaque assay on a weekly basis. No virus was detected in the first week of culture, confirming that active viral replication is not taking place at this time point. Iris explants from uninfected mice never showed any changes in the cellular monolayer (**Fig 9B**). After two weeks of culture, cytomegalic cells and foci of viral cytopathy were noted in several of the iris explants from latently infected mice (**Fig 9B**). Titration of culture supernatants on permissive fibroblasts was used to confirm the presence of reactivated infectious virus. MCMV reactivation was frequently observed in iris and choroid cultures, and rarely in corneal cultures (**Fig 9C**). These data provide unequivocal evidence that MCMV can establish latency in the eye, and highlight the eye as a reservoir of reactivating, infectious virus in immunocompetent hosts.

## Discussion

MCMV infection in mice is a reliable model for HCMV infection and considerable data on pathogenesis and disease in humans have been derived from the MCMV mouse model. We report here that systemic infection with MCMV in immunocompetent mice induces ocular inflammation in the form of uveitis (iritis, cyclitis and choroiditis), which is characterised by an acute replicative viral phase lasting 7–10 days and a chronic, granulomatous inflammatory phase which persists long-term, even when replicating virus is no longer detectable in eye tissues. Although no discernible replicating virus was detected in the retina, unexpected and sustained inflammatory changes were noted in this neural compartment. Notably, latent infectious virus could be readily reactivated from explanted uveal tissues, indicating that cytomegalovirus establishes an unexpected reservoir of virus in the eye of immunocompetent hosts. These findings provide support for the notion that in situations of immunosuppression, the presence of virus in the eye is unlikely to require spread of reactivating virus from non-ocular tissues and highlight the fact that in immunocompetent hosts sustained inflammatory changes can be elicited by infection.

Based on these data, several general observations relevant to host-viral interaction can be made. Firstly, the characteristic pathology of myeloid cell aggregates, which contain virally infected cells within focal areas located around vessels, resemble similar, if larger, lesions in the lung [31]. The nature of the tissue pathology induced in the iris by CMV is also reminiscent of damage in several other tissues, in that vasculitis is a prominent component of the inflammatory response [32–35].

Inflammation in the retina after systemic infection is similar to data obtained from studies in the brain [15, 35–37], with the notable difference that brain inflammation has only been reported either (i) after direct infection of the brain or (ii) after systemic infection of severely immunosuppressed or immune-incompetent hosts, and concomitantly with overt viral infection. Indeed, in mouse models, systemic infection leads to rapid infection of the brain parenchyma, as well as meningeal tissue (equivalent to retina and uveal tract respectively in the eye) in neonatal mice and in severely immunocompromised mice, but not in adult immunocompetent mice [15, 37–39]. These studies suggest that in adult mice the blood-brain barrier and/or immune-mediated mechanisms prevent intracerebral spread of MCMV. By contrast, in neonatal mice, where both the blood-brain barrier and the immune system are not fully developed, MCMV can infect neural cells [35, 37]. Similar control mechanisms may prevent infection of the retina; while we found that in immunocompetent mice MCMV infection was readily detected in choroidal cells which abut the retinal pigment epithelial (RPE) component of the blood-retinal barrier, infection of retinal parenchymal cells was not observed by the methods used here. Consistent with these findings, in humans, retinal pathology is observed in the severely immunocompromised [40]. Notably, overt and sustained inflammation (vasculitis and lymphocyte infiltration) was noted in the retina of mice after systemic viral infection and in the absence of discernible viral replication.

Our results provide evidence that CMV infection leads to sustained inflammatory changes in the eye and suggest that immunocompetent CMV-seropositive individuals may experience long-lasting low grade ocular inflammation. HCMV infection or reactivation is generally regarded as being asymptomatic in healthy individuals. This assumption is coming under increased scrutiny, with HCMV infection of apparently immunocompetent individuals reported to cause disease in organs including the gastrointestinal tract, the central nervous system and the eye [10]. In recent years, HCMV has been increasingly implicated in anterior uveitis and corneal endotheliitis [41–45]. In support of HCMV being the etiologic agent in these conditions, some patients respond to antiviral drugs [42, 44]. Many clinicians remain sceptical about the relevance of HCMV in the aetiology of anterior segment disease and therefore it is likely that the incidence of disease is underestimated, resulting in inappropriate treatment which can severely compromise vision. Our data provide evidence for the role of CMV infection in these diseases. OCT analyses performed after day 60 pi in our mouse model showed that the on-going inflammatory changes detected by immunohistology and FACS analyses could not be detected by OCT (**S2 Fig**), indicating that this technology is not sufficiently sensitive to monitor relevant inflammatory changes. Notably, the ongoing inflammation observed following CMV infection may predispose to other conditions, including age-related macular degeneration [46].

Our current study also addresses the important question as to which is the initial cell to be infected. Notably, the nature of the initially infected cell is likely to have relevance to the cellular source of latent infection. As shown in this study, endothelial cells in the iris are the first cells in which virus is detected; this is followed by infection of perivascular cells, including pericytes. Importantly, the areas of focal infection are associated with patches of vascular occlusion (vasculitis). As the virus is contained in foci around the vessels during the productive stage, and this remains the site where eventually viral gene expression is shut off, it is likely that uveal endothelial cells, and/or the associated resident myeloid cells, become the source of latent infection. Other possibilities are uveal tract stromal fibroblasts. Since the choroid tissue samples used in the reactivation assays also contain RPE, the involvement of RPE cells in supporting latent infection cannot be excluded. In previous studies, liver sinusoidal endothelial cells, as well as myeloid cells have been identified as potential sites of initial infection and latency [47]. As is well known, CMV is a superbly opportunistic virus with the potential to infect many cell types, and this may explain why the virus remains latent in such a large proportion of the population, although requiring a sustained inflationary T cell response to prevent the virus from reactivating and causing pathology *in vivo* [48]. Previous studies, using models where the virus was delivered directly in the eye have established that ocular compartments are permissive to CMV infection [24, 49]. Our studies have expanded the previous body of knowledge and provide novel evidence that systemic CMV infection can lead to viral replication in specific eye compartments, namely the anterior segment and choroid, but not the retina. Unlike previous studies, we have also shown that infection in the eye occurs in immunocompetent hosts. Importantly, our data demonstrate that systemic CMV infection of immunocompetent hosts leads to sustained inflammation in the eye, including the neural retina.

Retinal tissue is permissive to CMV infection as demonstrated by intraocular delivery of virus *in vivo*, as well as infection of retinal pigment epithelial cells *in vitro* [24, 50]. Clearly, in the setting of a functional immune system and an intact blood retinal barrier, as modeled in our current studies, the virus does not appear to be able to infect the retina. Despite the lack of detectable infection, an inflammatory response is elicited in the retina, which includes the presence of virus-specific CD8^+^ T cells and the formation of T_RM_ populations. T_RM_ are non-circulating subsets of memory T cells that mediate localized protective immunity to site-specific infections. They mount potent recall responses and accelerate the control of pathogens, especially at barrier sites.

At mucosal sites, such as the salivary gland, the formation of MCMV-specific T_RM_ CD8^+^ T cells can occur independently of viral replication, and this population can be supplemented in an ongoing manner by circulating T_RM_ [51]. The presence of MCMV-specific T_RM_ in the retina is more difficult to reconcile given the absence of viral replication noted in this tissue and the fact that the retina is protected by a tight retinal blood barrier. Two possibilities come to mind. The retina is a highly vascularized tissue and thus in one scenario it is possible that T_RM_ exit the vasculature and access the retinal parenchyma. The signals that drive egress of virus-specific T_RM_ from the retinal vasculature to the parenchyma remain to be identified. Alternatively, it is possible that the deposition of T_RM_ in retinal tissue is driven by viral antigen presented to these T cells independently of local viral replication. In such a situation it is possible that viral antigens are phagocytosed by microglia during the process of ‘cleaning’ up lysed MCMV infected cells in the anterior segment and/or retinal pigment epithelium and that these cells then present viral peptides. Whether the inflammatory milieu induced by infection is sufficient to cross-present these viral peptides in an immunogenic manner that stimulates local T cell proliferation and T_RM_ formation remains unknown. Thus, the cues required for the generation of retinal T_RM_, their functionality and role in protective immunity to CMV, as well as to potential collateral damage responses are important considerations that require further investigation.

In summary, the role of micro-organisms that inhabit and establish ‘residency’ in various tissues has received much attention recently because of the profound impact that they have on an extensive range of functions, including immunological, metabolic and hormonal responses. The microbiota of the eye remains poorly characterized and the impact of pathogens that reside in this tissue is largely unknown. The current findings clearly identify the eye as a reservoir for a common viral pathogen, and provide support for the notion that viral infection of ‘privileged sites’ may be a general phenomenon. Importantly, the ongoing inflammation observed in the eye following MCMV infection of immunocompetent hosts is unexpected and highlights the need to consider CMV as a pathogen capable of inducing long-lived inflammatory sequelae in the eye, including the neural retina.

## Materials and methods

### Mice

Inbred female BALB/c mice at 8–10 weeks of age were obtained from the Animal Resource Centre (Perth, Western Australia) and kept in specific pathogen free conditions.

### MCMV infection

Mice were infected by intraperitoneal administration of 1x10^4^ plaque-forming units of salivary gland propagated virus stock of MCMV diluted in phosphate-buffered saline (PBS) containing 0.05% fetal bovine serum. The viruses used were: MCMV-K181-Perth [52], MCMV-K181-Perth-LacZ [29], and MCMV-K181-Perth-mCherry. The mCherry virus was constructed by inserting the DNA sequence for the mCherry fluorescent protein in frame with C-terminus of IE1. A self-cleaving 2A peptide sequence separates the IE1 and mCherry sequences allowing for simultaneous expression of mCherry and IE1.

### Histology

Mice were euthanized and eyes removed and fixed in Davidson’s fixative for 12hr and stored in 10% neutral buffered formalin. Tissue sections of 5 μm were cut and stained with hematoxylin and eosin. X-gal staining was performed by freezing eyes in Tissue-Tek OCT compound (Sakura Finetek USA, Torrance, CA), and 7 μm sections cut. Staining of sections with X-gal was performed as described.

### Preparation of whole-mounts and microscopy

Mice were euthanized, and eyes collected and fixed in 2% paraformaldehyde for 1 hr before transferring into cold PBS. For staining of endothelial or pericyte markers eyes were fixed in ice cold methanol. After dissection of the various compartments of the eye (choroid, retina, iris) the tissues were incubated in 20 mM EDTA at 37°C for 30 minutes. Tissues were then incubated in a solution containing 0.3% Triton-X, 2% bovine serum albumin and 10% of normal goat serum in PBS at room temperature for 30 min. Tissues were incubated with the primary antibody overnight at 4°C followed by incubation with the secondary antibody at room temperature for 1 hr. Detection of the immediate-early (IE1) protein of MCMV was performed with the 6/58/1 monoclonal antibody [53]. Monoclonal antibodies specific for the following cell surface markers were used to stain tissue sections: MHC-class II (clone M5/114), CD45 (clone 30F11), CD31 (clone Mec 13.3), PDGFRβ (clone APB5), F4/80 (clone BM8), CD4 (clone RM4-5), CD8 (clone 53–6.7), IBA1 (Wako Pure Chemicals Industry, Osaka, Japan), CollagenIV (Biorad-2150-1470). Slides were counterstained with Hoechst to visualize nuclei. Assessment of stained specimens was performed using an epifluorescence microscope (Olympus BX60 microscope: Olympus, Tokyo, Japan) or a Nikon C2 Upright Confocal microscope.

### Viral quantification

Organ were collected and homogenized in cold Minimum Essential Medium (MEM) supplemented with 2% neonatal calf serum (NCS). After homogenisation insoluble material was removed by centrifugation (300 g for 15 min at 4°C) and the resulting supernatants were stored at -80°C. Viral titres were determined by absorbing serial dilutions of organ homogenates on confluent monolayers of M2-10B cells in 24-well tissue culture trays for 1hr at 37 ^o^C. The homogenate was removed by aspiration and cells overlaid with 1 ml of MEM 2% NCS containing 0.7% w/v carboxymethylcellulose and incubated for 4 days at 37 ^o^C. Plates were stained with a solution of 4% formaldehyde and 0.5% Methylene blue and plaques counted.

### Real time PCR method

The viral load in different compartments of the eye was determined by real-time quantitative PCR (qPCR). Briefly, eyes were dissected and DNA extracted from the iris/ciliary body, choroid/sclera and retina using the DNeasy Blood and Tissue Kit (Qiagen). Iris/ciliary body and choroid/sclera were pooled from 5 mice while retinas from both globes were pooled for each mouse. qPCR was performed with 100ng of purified DNA, glycoprotein B (gB) specific primers (F: ttggctgtcgtctagctgttt and R: taaggcgtggactagcgataa) and the Sso Advanced Universal SYBR Green system (Biorad). Serial dilutions of a synthesized MCMV gB sequence were used to generate a standard curve.

The presence of MCMV mRNA was determined by isolating total RNA from dissected compartments of the eye using the PureLink RNA kit (Ambion) according to the manufacturer’s protocols. Briefly, tissue was collected and placed directly into lysis buffer containing β-mercaptoethanol and homogenised using the Tissue Lyzer II (Qiagen) for 3 x 30 seconds at 23 Hz. The resulting lysate was passed through a PureLink RNA column, treated with DNase I, and eluted in RNase free water. The purity and quantity of RNA was assessed by a BioPhotometer (Eppendorf). The expression of IE1 and gB viral mRNA was determined using a two-step RT-qPCR assay; the expression of IE1 and gB was compared with that of ribosomal protein L32 mRNA. First, cDNA was generated from 2 μg of total RNA using Random primers and M-MLV Reverse Transcriptase (Promega). The cDNA samples were then used in the qPCR assay as described above using IE specific primers used (F: tgacttaaactccccaggcaa and R: taggtgaggccatagtggcag) or gB primers indicated above.

### Flow cytometry

Single cell suspensions of retinal tissue and iris/ciliary body were prepared at the indicated time points after infection. Briefly, eyes were dissected to separate the anterior from the posterior segment. The anterior segment was dissected further to yield the iris and the ciliary body while the posterior segment was dissected to separate the retina from the choroid/sclera. Retinas from both globes were pooled for each mouse. The iris and ciliary body tissue samples were pooled from multiple mice (up to five/group) before all tissue was minced and digested in a mixture of 10μg/ml Liberase (Roche, Germany, Cat No #05401119001) and 10μg/ml DNAse I (Sigma, USA) in PBS for 40 minutes at 37°C. The resulting single cell preparations were stained with antibodies specific for CD45 (30F11), CD11b (M1/70), CD3 (145-2C11), CD4 (RM4-5), CD8 (53–6.7), NKp46 (29A1.4), CD11c (HL3), CD19 (6D5), CD64 (X54-5/7.1), F/480 (BM8), MHC-II (M5/114), Ly6C (AL.21), Ly6G (1AB); CD69 (H1.2F3), CD103 (M290). Virus-specific CD8^+^ T cells were identified using tetramers for H-2Ld-YPHFMPTNL MCMV-IE1 from Immuno ID Tetramers (Melbourne, Victoria). Antibodies were obtained from BD Biosciences, BioLegend, or eBioscience. Fixable viability stain 620 (BD Biosciences) was used for live/dead discrimination. Samples were analysed using an LSRFortessa X-20 instrument (BD Biosciences). The gating strategies used to identify immune cell populations in the iris and retina are shown in **S3 Fig**. All data analysis was performed using the FlowJo software package (FlowJo, LLC).

### Live ocular imaging

Fluorescent images were obtained using a Phoenix Micron IV Retinal Imaging Microscope (Phoenix Research Laboratory San Ramon, CA-USA). Fluorescent images were captured after anesthetising mice with a mixture of ketamine and xylazine (Troy Laboratories, Australia). Genta-Gel lubricant eye gel was used on the eye surface to prevent drying of the cornea. Vessels were visualised by subcutaneous injection of 20 μl of 10% sodium fluorescein solution (Alcon, Australia).

OCT images of the anterior segment were obtained using an EnvisuR2200 SD-OCT system with a 12mm-telocentric lens (Leica Microsystems). Anterior segment OCT analysis was performed in the absence of anaesthesia with lubricant eye drops (Refresh Tears Plus, Allergan, USA) applied throughout the procedure to maintain corneal clarity. OCT fundus images of the retina were acquired using the EnvisuR2200 SD-OCT system with the mouse retina lens. The mice were anesthetized as described above. Vessel size on the fundus images were measured using the caliper function available in Bioptigen OCT software.

### Tissue explants

Eyes from infected mice were removed between day 70 and 170 pi and iris/ciliary body, cornea, retina and choroid dissected. Each component of the eye was placed in a well of a 48 well tray and 100 μl of minimum essential medium (MEM) containing 10% FCS and 7g/L carboxymethyl cellulose added. After 24 hr of culture at 37°C, 500 μl of MEM supplemented with 10% FCS was added to each well. Medium was collected from each well every 7 days thereafter and assayed for the presence of MCMV by plaque assay for up to 35 days. Reactivated infectious virus was detected between day 7 and day 14 of culture.

### Ethics statement

All experiments were performed in accordance with the recommendations in the Australian Code of Practice for the Care and Use of Animals for Scientific Purposes and the National Health and Medical Research Council of Australia Guidelines and Policies on Animal Ethics. Experiments were approved by the Animal Ethics Committee of the University of Western Australia (Animal Ethics Research Protocol RA/3/100/1094) and the Harry Perkins Institute of Medical Research Animal Ethics Committee (AEC Project Number AE031/2015).

### Statistical analysis

All data except for measurements of vessels size data was assessed using a two-tailed Mann-Whitney test. Changes in vessel diameter were assessed using a one-way ANOVA test with Dunn’s correction. Statistical tests were performed using the statistical software package InStat (GraphPad Software, San Diego California USA).

## Supporting information

S1 Fig Detection of MCMV RNA and genomes and by RT-PCR. **(A)** BALB/c mice were infected with MCMV and the presence of viral DNA in the indicated eye compartments was quantified by RT-PCR at day 5 or 9 pi (n = 5). ND = not detected, limit of detection 50 copies/reaction. **(B)** Relative expression of IE1 or gB mRNA in the indicated compartments of the eye relative to L32 mRNA from uninfected mice or at day 5pi (n = 5). BLD = below limit of detection.
(TIF)

S2 Fig OCT analysis of eyes in latently infected mice. SD-OCT images of anterior chamber from an uninfected mouse, or two MCMV-infected mice at 78 day pi. At this time point the eyes of mice harbouring a latent MCMV show no sign of pathological features related to the viral infection.
(TIF)

S3 Fig Flow cytometry gating strategy. Immune cell populations localised to the iris or retina were identified by flow cytometry using the indicated gating strategy.
(TIF)
