# Supplementary figures and images for: Cytomegalovirus establishes a latent reservoir and triggers long-lasting inflammation in the eye
Source: PLoS Pathog. 2018 May 31;14(5):e1007040. doi: 10.1371/journal.ppat.1007040 (PMC5978784; doi:10.1371/journal.ppat.1007040)

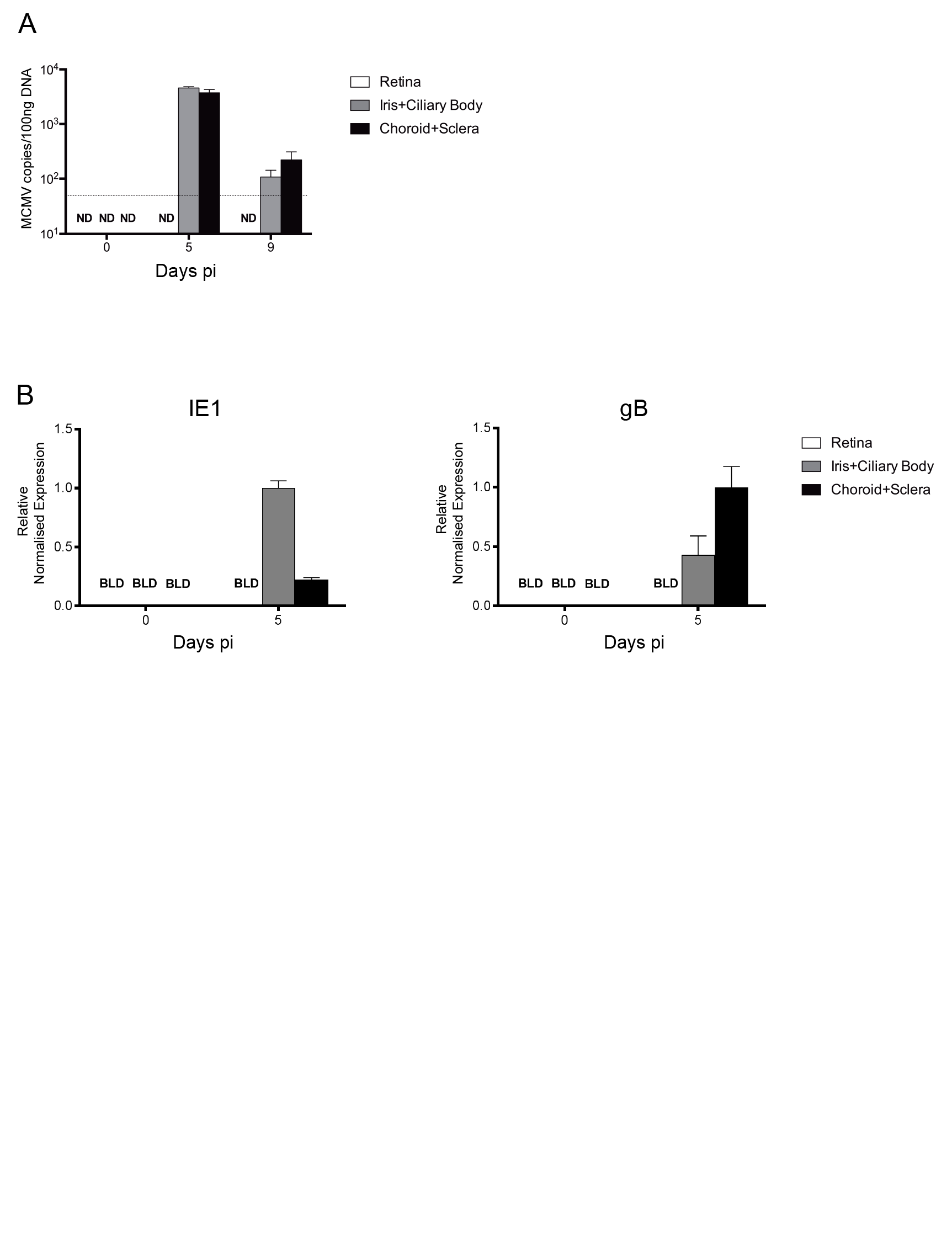

Supplement: S1 Fig — (A) BALB/c mice were infected with MCMV and the presence of viral DNA in the indicated eye compartments was quantified by RT-PCR at day 5 or 9 pi (n = 5). ND = not detected, limit of detection 50 copies/reaction. (B) Relative expression of IE1 or gB mRNA in the indicated compartments of the eye relative to L32 mRNA from uninfected mice or at day 5pi (n = 5). BLD = below limit of detection. (TIF) [file ppat.1007040.s001.tif]

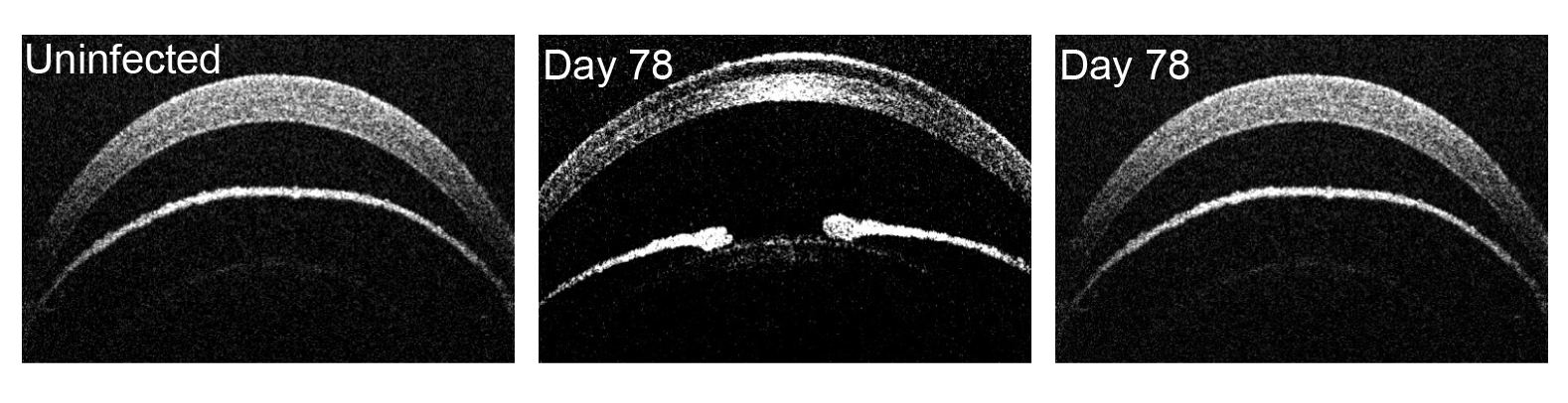

Supplement: S2 Fig — SD-OCT images of anterior chamber from an uninfected mouse, or two MCMV-infected mice at 78 day pi. At this time point the eyes of mice harbouring a latent MCMV show no sign of pathological features related to the viral infection. (TIF) [file ppat.1007040.s002.tif]

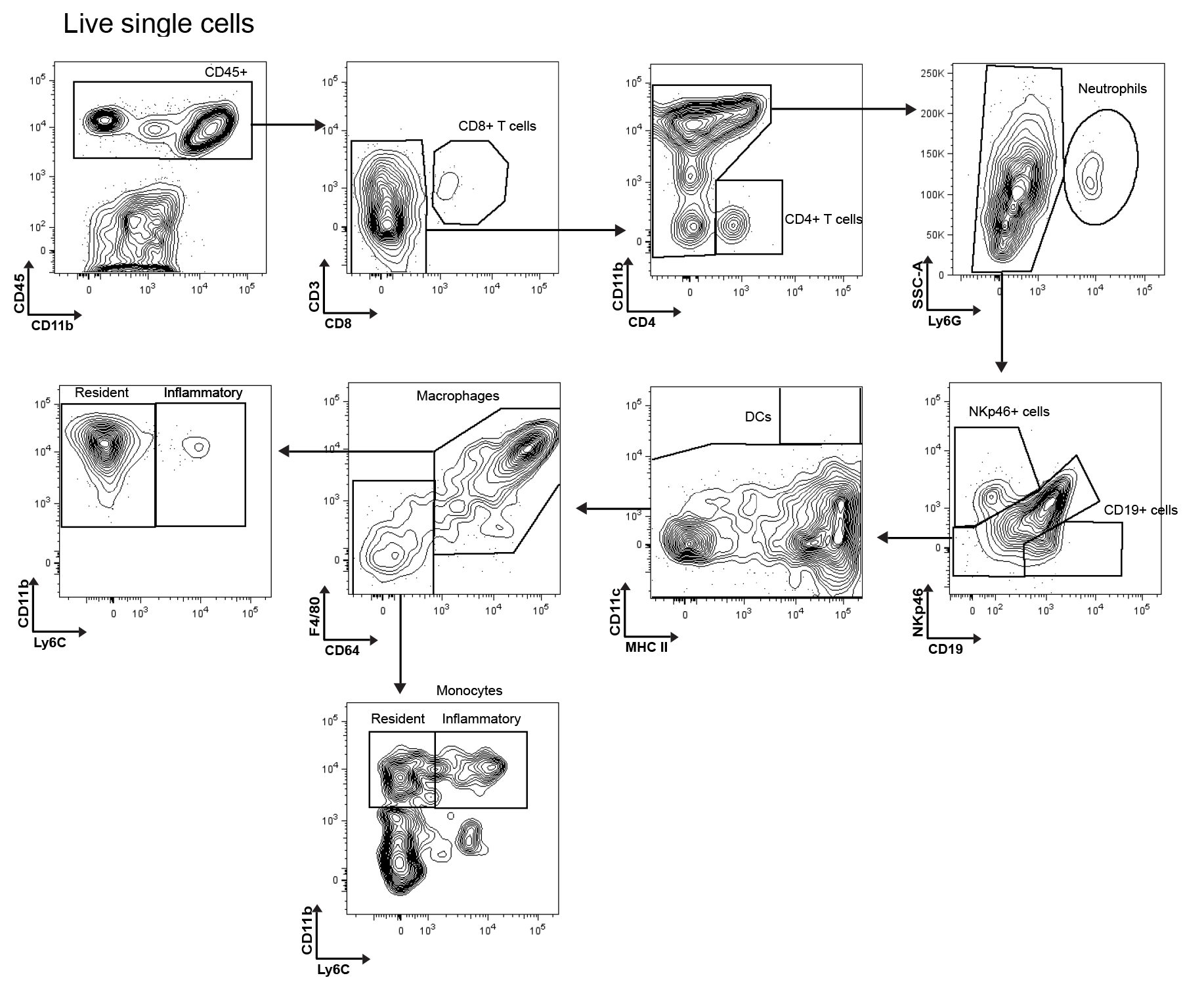

Supplement: S3 Fig — Immune cell populations localised to the iris or retina were identified by flow cytometry using the indicated gating strategy. (TIF) [file ppat.1007040.s003.tif]
